# Diagnostic ability of vessel density measured by spectral-domain optical coherence tomography angiography for glaucoma in patients with high myopia

**DOI:** 10.1038/s41598-020-60051-0

**Published:** 2020-02-20

**Authors:** Kwanghyun Lee, Kyung Joo Maeng, Joo Yeon Kim, Heon Yang, Wungrak Choi, Sang Yeop Lee, Gong Je Seong, Chan Yun Kim, Hyoung Won Bae

**Affiliations:** Department of Ophthalmology, Severance Hospital, Institute of Vision Research, Yonsei University College of Medicine, Seoul, Korea

**Keywords:** Diagnostic markers, Eye manifestations

## Abstract

Although early glaucoma detection is important to prevent visual loss due to disease progression, its clinical diagnosis in highly myopic eyes is still difficult. Many studies using optical coherence tomography (OCT) angiography (OCTA) reported decreased vessel density (VD) in glaucomatous eyes compared to normal eyes. We evaluated the diagnostic ability of peripapillary VD and macular VD measured by OCTA, comparing them with conventional valuables such as peripapillary retinal nerve fibre layer (RNFL) thickness and macular ganglion cell-inner plexiform layer (GCIPL) thickness measured by OCT. We also calculated the average VD ratio (VDR) (average outer macular VD/average inner macular VD), superior VDR (superior outer macular VD/average inner macular VD), and inferior VDR (inferior outer macular VD/average inner macular VD). Totally, 169 eyes from 169 subjects were enrolled. Among OCTA measurements, the best diagnostic parameters were average VDR (AUROC: 0.852 and 0.909) and inferior VDR (AUROC: 0.820 and 0.941) in nonhighly and highly myopic eyes, respectively. Inferior VDR showed better diagnostic ability than most of the other OCT measurements including peripapillary RNFL thickness and macular GCIPL thickness in highly myopic eyes. Accordingly, OCTA measurements can be useful for diagnosing glaucoma in highly myopic eyes, especially when using calculated indices such as average VDR or inferior VDR.

## Introduction

Glaucoma is a progressive optic neuropathy characterised by retinal ganglion cell loss and visual field (VF) change^1^. Early detection of glaucoma is important to prevent visual loss caused by disease progression. However, clinical diagnosis of glaucoma in myopic eyes is often difficult^2^. The evaluation of the optic disc is especially difficult in highly myopic eyes because of considerable morphologic variations^3^. The retinal nerve fibre layer (RNFL) and ganglion cell-inner plexiform layer (GCIPL) can also be mistaken for glaucoma as they increase in thinness as the axial length increases^4,5^.

Spectral-domain optical coherence tomography (SD-OCT) has grown in importance in diagnosing glaucoma, allowing clinicians to assess structural changes in the optic disc and macula^6–10^. Further, recent studies have shown that peripapillary vessel density (VD) and macular VD, as measured by OCT angiography (OCTA), in glaucoma eyes are reduced^11,12^. Interestingly, a recent study reported that peripapillary VD well correlated with VF defects in both non-highly and highly myopic eyes^13^. Therefore, peripapillary VD or macular VD could be useful for glaucoma detection in highly myopic eyes, despite reports that macular VD measurements are not better than macular GCIPL thickness measurements for glaucoma detection in non-highly myopic eye^14^. To the best of our knowledge, few studies have compared the diagnostic ability of peripapillary VD and macular VD for glaucoma detection in highly myopic eyes.

In this study, we compared the diagnostic ability of peripapillary VD and macular VD for glaucoma and compared it with that of peripapillary RNFL thickness and macular GCIPL thickness in both highly and nonhighly myopic eyes.

## Results

A total of 241 eyes were examined, of which 16 eyes were excluded because of the presence of other eye diseases, such as diabetic retinopathy, which could affect VD or poor image quality. We also excluded three eyes with a history of ocular surgery. Fifty-three eyes in which spherical equivalent exceeded −0.5 D were excluded as well. A total of 169 eyes from 169 subjects were enrolled. Subjects were divided into two groups: highly myopic eyes (n = 60) and non-highly myopic eyes (n = 109), with 86 normal eyes and 83 open-angle glaucoma eyes. Of the 54 eyes with non-highly myopic glaucoma, 18 eyes had superior, 12 eyes had inferior, and 24 eyes had both superior and inferior hemifield VF defects. Among the 29 eyes with highly myopic glaucoma, 15 eyes had superior, three eyes had inferior, and 11 eyes had both superior and inferior hemifield VF defects (P = 0.231).

As expected, the MD of the VF was significantly worse in glaucomatous eyes in both groups (Table 1). However, other variables were not significantly different between the normal and glaucomatous eyes in both groups. Age, spherical equivalent, and axial length differed between nonhighly and highly myopic eyes (Table 1).

**Table 1:** Characteristics of subjects.

	Nonhighly myopic eyes (n = 109)			Highly myopic eyes (n = 60)			
	Normal (n = 55)	Glaucoma (n = 54)	P*	Normal (n = 31)	Glaucoma (n = 29)	P^†^	P^‡^
Age (y)	45.9 ± 13.4	50.2 ± 11.0	0.070	37.7 ± 12.4	42.7 ± 13.5	0.142	**<0.001**
Sex (M:F)	24:31	34:20	0.067	15:16	16:13	0.789	0.975
HTN (Y:N)	9:46	11:43	0.770	5:26	3:26	0.781	0.533
DM (Y:N)	4:51	2:52	0.691	2:29	1:28	0.999	>0.999
SE (D)	−3.4 ± 1.8	−3.2 ± 1.8	0.505	−8.0 ± 2.1	−8.7 ± 1.8	0.160	**<0.001**
AL (mm)	25.4 ± 1.3	25.2 ± 1.5	0.318	27.3 ± 1.6	27.6 ± 1.2	0.378	**<0.001**
MD (dB)	−0.9 ± 1.5	−9.9 ± 6.5	**<0.001**	−1.5 ± 2.0	−11.8 ± 7.3	**<0.001**	0.356

M: male, F: female, Y: yes, N: no, HTN: hypertension, DM: diabetes mellitus, SE: spherical equivalent, AL: axial length, MD: mean deviation

The data are given as mean ± SD, unless otherwise specified. Values significant at P < 0.05 are indicated in bold.

*Value for comparison between normal and glaucomatous eyes in the highly myopic group.

^†^Value for comparison between normal and glaucomatous eyes in the nonhighly myopic group.

^‡^Value for comparison between highly and nonhighly myopic groups.

### OCT and OCTA measurements

The average and inferior peripapillary RNFL thicknesses significantly differed between normal and glaucomatous eyes in both highly and nonhighly myopic eyes (all P < 0.05, Table 2). The average peripapillary RNFL thickness was not different between nonhighly myopic eyes and highly myopic eyes, but the inferior peripapillary RNFL thickness was different after adjusting for age (Table 2). The minimum and inferotemporal macular GCIPL thicknesses were significantly different between normal and glaucomatous eyes in both nonhighly myopic eyes and highly myopic eyes (all P < 0.05, Table 2). Macular GCIPL thickness was more in nonhighly myopic eyes than in highly myopic eyes after adjusting for age (Table 2, Supplementary Table S1).

**Table 2:** Peripapillary vessel density and macular vessel density measured by optical coherence tomography angiography.

	Nonhighly myopic eyes (n = 109)			Highly myopic eyes (n = 60)				
	Normal (n = 55)	Glaucoma (n = 54)	P*	Normal (n = 31)	Glaucoma (n = 29)	P**	P^†^	P^‡^
**Main RNFL thickness parameters (*μ*m)**								
Average	86.5 ± 10.4	69.9 ± 12.9	**<0.001**	85.5 ± 9.8	69.3 ± 8.9	**<0.001**	0.800	0.118
Inferior	106.2 ± 20.3	75.1 ± 19.0	**<0.001**	102.2 ± 19.1	66.7 ± 13.4	**<0.001**	0.149	**0.023**
**Macular GCIPL parameters (*μ*m)**								
Minimum	74.5 ± 7.7	56.6 ± 9.4	**<0.001**	66.8 ± 10.8	54.8 ± 6.6	**<0.001**	**0.017**	**<0.001**
Inferotemporal	77.5 ± 8.1	60.9 ± 10.6	**<0.001**	72.9 ± 6.7	57.0 ± 7.8	**<0.001**	**0.037**	**0.004**
**Vessel density – optic disc scan (mm**^**−1**^**)**								
Average – Full	16.5 ± 2.2	15.2 ± 2.2	**0.003**	15.8 ± 3.7	13.6 ± 3.7	**0.025**	**0.046**	**0.001**
Average – Outer	17.4 ± 2.3	15.9 ± 2.4	**0.002**	16.3 ± 4.1	14.1 ± 4.0	**0.042**	**0.017**	**<0.001**
Average – Inner	15.5 ± 3.2	14.3 ± 2.8	**0.049**	15.7 ± 3.6	13.4 ± 4.1	**0.022**	0.615	0.108
Average – Centre	2.5 ± 2.8	2.1 ± 2.4	0.414	4.2 ± 4.2	2.5 ± 2.7	0.063	0.051	**0.023**
**Vessel density – macular scan (mm**^**−1**^**)**								
Average – Full	17.1 ± 1.8	15.5 ± 2.2	**<0.001**	17.3 ± 1.5	14.9 ± 2.6	**<0.001**	0.504	0.071
Average – Outer	17.4 ± 1.7	15.6 ± 2.4	**<0.001**	17.6 ± 1.6	14.8 ± 2.7	**<0.001**	0.494	0.054
Inferior Outer	16.9 ± 2.2	13.7 ± 3.3	**<0.001**	17.3 ± 2.2	11.9 ± 3.8	**<0.001**	0.304	**0.031**
Average – Inner	17.0 ± 2.2	16.9 ± 2.1	0.754	17.3 ± 1.6	16.3 ± 2.5	0.085	0.732	0.376
Inferior Inner	16.8 ± 2.6	16.3 ± 2.5	0.382	17.5 ± 1.7	14.8 ± 3.5	**0.001**	0.420	0.153
Average – Centre	8.2 ± 3.2	8.2 ± 3.1	0.939	8.9 ± 2.9	8.1 ± 2.9	0.329	0.508	0.920
Average VDR	1.0 ± 0.1	0.9 ± 0.1	**<0.001**	1.0 ± 0.1	0.9 ± 0.1	**<0.001**	0.305	**0.035**
Inferior VDR	1.0 ± 0.2	0.8 ± 0.2	**<0.001**	1.0 ± 0.1	0.7 ± 0.2	**<0.001**	0.133	**0.016**
Superior VDR	1.0 ± 0.1	1.0 ± 0.1	**<0.001**	1.0 ± 0.1	1.0 ± 0.1	0.059	0.347	0.875

VDR: vessel density ratio, RNFL: retinal nerve fibre layer, GCIPL: ganglion cell-inner plexiform layer, Average VDR: average outer macular vessel density/average inner macular vessel density, Inferior VDR: inferior outer macular vessel density/average inner macular vessel density.

The data are given as mean ± SD. Values significant at P < 0.05 are indicated in bold.

*Value for multiple comparisons between normal and glaucomatous eyes in the highly myopic group.

**Value for multiple comparisons between normal and glaucomatous eyes in the nonhighly myopic group.

^†^Value for multiple comparisons between highly and nonhighly myopic groups (t-test).

^‡^Values for multiple comparison between highly and nonhighly myopic groups after adjusting for age (linear regression).

The average peripapillary VD at the inner, outer, and full areas significantly differed between normal and glaucomatous eyes in both nonhighly and highly myopic eyes (all P < 0.05, Table 2). After adjusting for age, the peripapillary VD at the centre, outer, and full areas statistically decreased in highly myopic eyes than in nonhighly myopic eyes (all P < 0.05, Table 2).

The average macular VD at the outer and full areas significantly differed between normal and glaucomatous eyes in both nonhighly and highly myopic eyes (all P < 0.05, Table 2). In contrast, average inner and centre macular VDs were not statistically different in both nonhighly myopic eyes and highly myopic eyes (all P > 0.05, Table 2).

The average VDR and inferior VDR were statistically different between normal and glaucomatous eyes in both nonhighly and highly myopic groups (all P < 0.05, Table 2), but the superior VDR was statistically different between the normal and glaucomatous eyes only in nonhighly myopic eyes (P < 0.001), but not in highly myopic eyes (P = 0.059, Table 2). In comparison between the nonhighly myopic eyes and highly myopic eyes, the average VDR and inferior VDR were statistically different (P = 0.033 and P = 0.015, respectively), but the superior VDR was not (P = 0.870, Table 2).

### Diagnostic ability of OCT and OCTA measurements

**Figure 1:**
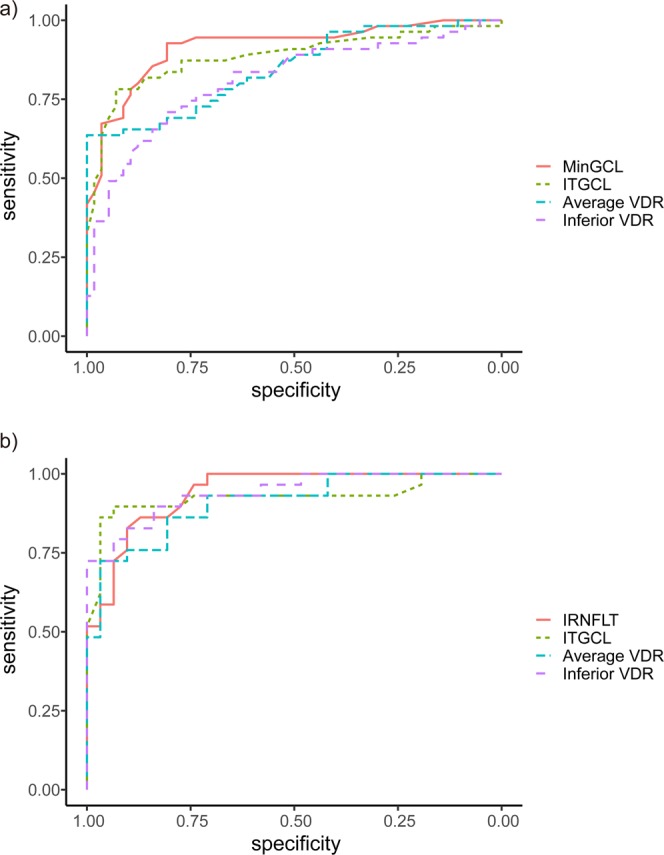
Receiver operating characteristic curves of best parameters of optical coherence tomography (OCT) and OCT angiography in (**a**) nonhighly myopic eyes and (**b**) highly myopic eyes. MinGCL: minimum ganglion cell-inner plexiform layer (GCIPL) thickness, ITGCL: inferior temporal GCIPL thickness, VDR: vessel density ratio, Average VDR: average outer macular vessel density/average inner macular vessel density, Inferior VDR: inferior outer macular vessel density/average inner macular vessel density, IRNFLT: inferior retinal nerve fibre layer thickness.

The area under the receiver operating characteristic (AUROC)s and the sensitivities at 90% specificity of VD measurements from OCTA and thickness measurements from OCT that were calculated to differentiate glaucomatous eyes from normal eyes are shown in Table 3 and Supplementary Table S2. In nonhighly myopic eyes, the minimum GCIPL thickness and the average VDR had the best diagnostic ability among all OCT measurements and OCTA measurements, respectively. By DeLong test^15^, the diagnostic ability of average VDR was worse than that of minimum GCIPL thickness, in nonhighly myopic eyes (P = 0.009). In contrast, in highly myopic eyes, inferior RNFL and inferior VDR were the best parameters among OCT measurements and OCTA measurements, respectively, for glaucoma diagnosis. The diagnostic ability of inferior VDR in highly myopic eyes was comparable with that of the inferior RNFL thickness (P = 0.652, Fig. 1).

**Table 3:** AUROC curve values in nonhighly and highly myopic eyes among normal and glaucomatous eyes.

	Nonhighly Myopic Eyes (n = 109)		Highly myopic eyes (n = 60)		P*
	AUROC (95% CI)	Sensitivity at 90% specificity (%)	AUROC (95% CI)	Sensitivity at 90% specificity (%)	
**Main RNFL thickness parameters (*μ*m)**					
Average	0.831 (0.754–0.907)	61.1 (44.4–75.9)	0.887 (0.805–0.969)	63.7 (43.3–87.3)	0.217
Inferior	0.866 (0.796–0.936)	68.5 (46.9–86.1)	0.927 (0.866–0.988)	80.0 (46.7–96.7)	0.095
**Macular GCIPL parameters (*μ*m)**					
Minimum	0.917 (0.863–0.971)	77.8 (56.1–93.8)	0.855 (0.755–0.956)	48.5 (10.0–87.3)	0.318
Inferotemporal	0.881 (0.812–0.949)	77.8 (59.3–88.9)	0.925 (0.848–1.000)	90.0 (73.3–100.0)	0.490
**Vessel density – optic disc scan (mm**^**−1**^**)**					
Average – Full	0.706 (0.606–0.806)	26.1 (5.6–55.6)	0.712 (0.576–0.848)	31.7 (0.0–70.0)	0.861
Average – Outer	0.705 (0.604–0.806)	25.9 (6.6–54.1)	0.694 (0.556–0.833)	16.7 (0.0–66.7)	0.998
Average – Inner	0.631 (0.523–0.738)	16.9 (0.0–40.7)	0.669 (0.531–0.808)	33.9 (3.3–60.0)	0.626
Average – Centre	0.559 (0.449–0.670)	20.5 (9.4–33.8)	0.624 (0.481–0.768)	16.7 (5.0–40.0)	0.402
**Vessel density – macular scan (mm**^**−1**^**)**					
Average – Full	0.702 (0.603–0.800)	33.3 (18.5–48.2)	0.763 (0.643–0.884)	46.7 (20.0–70.0)	0.323
Average – Outer	0.731 (0.636–0.826)	37.0 (22.2–52.8)	0.809 (0.700–0.917)	50.0 (26.7–80.0)	0.206
Inferior Outer	0.790 (0.704–0.877)	51.9 (35.2–69.0)	0.890 (0.809–0.972)	73.3 (48.7–93.3)	0.070
Average – Inner	0.523 (0.414–0.633)	10.7 (0.0–27.8)	0.596 (0.450–0.743)	26.7 (6.7–50.0)	0.330
Inferior Inner	0.566 (0.457–0.675)	13.0 (1.9–32.4)	0.757 (0.627–0.886)	60.0 (30.0–76.8)	**0.021**
Average – Centre	0.505 (0.396–0.615)	9.3 (0.0–25.9)	0.558 (0.411–0.705)	20.0 (3.3–46.7)	0.458
Average VDR	0.852 (0.781–0.923)	65.5 (52.7–76.4)	0.909 (0.837–0.981)	73.3 (53.3–93.3)	0.272
Inferior VDR	0.820 (0.739–0.902)	59.3 (40.7–77.8)	0.941 (0.886–0.996)	80.0 (60.0–96.7)	**0.017**
Superior VDR	0.695 (0.596–0.794)	29.6 (16.7–44.4)	0.664 (0.518–0.811)	44.8 (17.3–69.0)	0.733

AUROC: area under the receiver operating characteristics, CI: confidence interval, RNFL: retinal nerve fibre layer, GCIPL: ganglion cell-inner plexiform layer, VDR: vessel density ratio, Average VDR: average outer macular vessel density/average inner macular vessel density, Inferior VDR: inferior outer macular vessel density/average inner macular vessel density.

*Calculated by DeLong *et al*.’s method^15^. Values significant at P < 0.05 are indicated in bold.

When comparing the AUROCs of OCTA measurements between nonhighly and highly myopic eyes (Table 3, Supplementary Table S2), the inferior VDR had better diagnostic performance in highly myopic than in nonhighly myopic eyes (P = 0.012).

## Discussion

In this study, we determined the diagnostic ability of OCTA measurements and compared them with OCT measurements in nonhighly and highly myopic eyes. The average VDR and inferior VDR showed great diagnostic performance for glaucoma in both nonhighly and highly myopic eyes. In particular, inferior VDR was one of the best parameters among OCT and OCTA measurements for glaucoma detection in highly myopic eyes.

The effect of myopia on peripapillary VD has been reported^13,16^. Suwan *et al*. used 4.5 × 4.5-mm optic disc scan and reported a nonsignificant reduction in peripapillary VD in moderately to highly myopic eyes compared with a control group^16^. Akagi *et al*. also reported a statistically similar peripapillary VD in patients with primary open-angle glaucoma with and without high myopia using 3 × 3-mm optic disc scan^13^. The area used in both studies could be considered as peripapillary VD in the inner area that were not different between nonhighly myopic eyes and highly myopic eyes in our study. However, in our study, peripapillary VD measurements in the outer area were reduced in highly myopic eyes than in nonhighly myopic eyes; it was also reduced in glaucomatous eyes compared to normal eyes both in nonhighly myopic eyes and highly myopic eyes. These results suggest that axial elongation due to myopic change might affect outer area (3–6  mm) around optic disc, not inner area (<3  mm).

The comparison of macular VD between nonhighly and highly myopic eyes showed no significant differences. This result is consistent with that of other studies obtained using a different OCTA method^17^ and Heidelberg retinal flowmetry^18^. In contrast, some studies have shown that macular VD decreases in myopic eyes^19–22^. One reason for this difference in results may be because the subjects in each study have varying characteristics. Milani *et al*. studied the relationship between macular VD and the spherical equivalent and compared the VD between controls (refractive error, 0  ±  2 D) and highly myopic eyes (refractive error ≥ − 6 D, mean, −10.26 D)^22^. The mean spherical equivalent of subjects in our study and Wang *et al*.’s study was a refractive error of about −8 D^17^. Therefore, it may not be enough to reveal the relationship between myopia and macular VD. These results imply that although myopic changes can affect the macular VD, the effect would be much weaker than that induced by glaucoma changes.

While previous studies with OCTA used a 3 × 3-mm macular scan^23,24^, our study used a 6 × 6-mm scan. In a previous study using a 4.5 × 4.5-mm disc scan and a 3 × 3-mm macular scan, the optic disc scan showed better performance in diagnosing glaucoma compared to the macular scan^25^. However, a 6 × 6-mm scan can detect glaucoma-induced changes in the macula that may be missed when using a 3 × 3-mm scan. Additionally, a previous study compared the diagnostic ability of macular VD between a 6 × 6-mm scan and 3 × 3-mm scan, and the 6 × 6-mm scan showed better performance in diagnosing glaucoma^14^. Our study showed that in a 6 × 6-mm scan, the macular VD had a better or comparable diagnostic ability than peripapillary VD for glaucoma, not only in nonhighly myopic eyes but also in highly myopic eyes Interestingly, the diagnostic performance of the outer macular VD was better than that of the inner macular VD, and the outer peripapillary VD was similar to the inner peripapillary VD in diagnostic performance. These results could be explained by the anatomical characteristics of peripapillary RNFL and macular GCIPL. Since retinal nerve fibres extend from the optic disc to the peripheral retina and early glaucoma changes generally involve the inferior or superior area, the diagnostic performances of the outer and inner peripapillary VDs may not be different. Due to the anatomical characteristics of the macula, early changes in glaucoma usually involve the outer macula first, thus leading to different diagnostic abilities between outer and inner macular VDs. In this context, a wider area for OCTA measurements may have better diagnostic performance. In particular, some OCTA can produce a 8 × 8-mm macular scan^26^, and therefore, comparison of diagnostic performances between a 6 × 6-mm scan and a wider scan such as an 8 × 8-mm scan is possible.

Three parameters (Average, inferior, and superior VDRs) were presented using the OCTA measurements. The diagnostic performance of the average and inferior VDRs were better than that of other OCTA measurements in both nonhighly and highly myopic eyes. Previous studies have shown that OCTA measurements were susceptible to signal strength variations caused by cataract, vitreous opacity, etc.^27^. We assumed that while the macular VD was affected almost equally by signal strength at all locations, glaucoma changes affected macular VD differently according to location; therefore, the relative values between OCTA measurements would have a better performance. The average VD of the outer macular sector statistically decreased in both nonhighly and highly myopic eyes with glaucome, but that of the inner macular sector did not. This difference may be the reason these parameters perform better in diagnosing glaucoma. Although inferior RNFL thickness and inferotemporal GCIPL thickness showed excellent diagnostic performance similar to the average VDR and inferior VDR in highly myopic eyes, some ocular features such as macrodisc, large peripapillary atrophy, and posterior staphyloma, in highly myopic eyes, could decrease the diagnostic performance of OCT measurements^28,29^. In that case, OCTA parameters such as the average or inferior VDR could be used to diagnose glaucoma in highly myopic eyes. However, the diagnostic ability of the superior VDR was relatively low. This result could be explained by the lesser number of eyes with inferior hemifield VF defects, since the superior VDR would correlate with the inferior hemifield VF defects.

Another interesting finding of our study was the difference in diagnostic performance of OCTA measurements between highly and nonhighly myopic eyes. It is not clear why the inferior VDR showed better diagnostic performance in highly myopic eyes than in nonhighly myopic eyes. This can be due to the difference in distribution of spatial hemifield VF defects. The number of eyes with superior hemifield VF defect was relatively high in highly myopic eyes. Although statistically insignificant, this higher ratio of superior hemifield VF defects could lead to better diagnostic ability of inferior VDR in highly myopic eyes. A magnification effect due to myopia could also be a reason for these results. According to the Littman and Bennett formula, the true diameter of the fundus increases with increasing axial length^30^. Since the area measured by OCTA could be larger in highly myopic eyes than in nonhighly myopic eyes, the inferior inner macular VD measurements in highly myopic eyes may include the area corresponding to inferior outer macular VD measurements, with a better diagnostic ability for glaucoma in nonhighly myopic eyes. Similarly, the inferior outer macular VD measurements in highly myopic eyes may be able to analyse a wider area. Although the difference in the inferior outer macular VD between nonhighly and highly myopic eyes was not statistically significant, the AUROC of the inferior outer macular VD in highly myopic eyes was larger than that in nonhighly myopic eyes, which could result in better performance of the inferior VDR. However, the reason only the inferior sector was affected by magnification is still unclear.

There are some limitations to this study. First, the highly myopic group was younger than the nonhighly myopic group. It is believed that because of the characteristic features of the optic nerve head, more patients in the highly myopic group were referred to the glaucoma clinic earlier. However, since the age between normal eyes and glaucoma eyes was not significantly different in both groups, the diagnostic ability of OCT measurements is considered not to be affected by age. Next, in highly myopic glaucoma eyes, there were more superior hemifield VF defects. A study reported more superior hemifield VF defects in myopia NTG^31^, but some studies have reported different results^32,33^. In glaucomatous eyes with high myopia, were were unable to conclude whether the greater number of superior hemifield VF defects was a natural characteristic of myopic glaucoma or merely a characteristic of the participants in our study. Therefore, the results of our study, which reported excellent the diagnostic ability of inferior VDR, should be validated in large group studies. Finally, although we showed that the inferior VDR and average VDR have superior diagnostic ability for glaucoma detection, there might have been a few false negative cases by its definition. When analysing eyes only with inferior hemifield VF defects, the inferior VDR could be normal. Otherwise, average VDR could be normal, when analysing eyes with peripapillary RNFL defect without macular involvement. Therefore, further studies are needed to identify new parameters that exhibit superior diagnostic performance and minimum false negatives.

In conclusion, we found that outer macular VD measurements were better than other macular VD measurements and peripapillary VD measurements for glaucoma diagnosis. The average VDR and inferior VDR showed greater ability for glaucoma detection than OCTA measurements themselves in both nonhighly and highly myopic eyes. These parameters also showed a comparable diagnostic performance with OCT measurements in highly myopic eyes.

## Methods

### Study design and participants

This retrospective cross-sectional study was performed in accordance with the tenets of Declaration of Helsinki and was approved retrospectively by the Institutional Review Board at Yonsei University (4-2019-0371). The requirement for informed consent was waived due to the retrospective nature of the study. Data from the records of patients who visited the glaucoma clinic at Severance Hospital of Yonsei University from July 2017 to August 2019 were analysed. The complete medical history of all subjects, including presence/absence of diabetes mellitus and hypertension, was noted. All subjects underwent complete ophthalmic examinations, including slit-lamp biomicroscopy, intraocular pressure (IOP) measurement using Goldmann applanation tonometry, gonioscopy, dilated fundus exams, and standard automated perimetry in both eyes. Peripapillary RNFL thickness and macular GCIPL thickness were measured by spectral-domain OCT (Cirrus HD-OCT 5000; Carl Zeiss Meditec, Dublin, CA), and macular VD and peripapillary VD were obtained from OCTA imaging (Cirrus-AngioPlex, version 10.0; Zeiss Medical Technology, Dublin, CA). Axial length was measured using IOL Master 700 (Carl Zeiss Meditec). Only participants >18 years of age with open angles on gonioscopy were included.

Normal eyes were required to have an IOP of ≤21  mmHg with no history of elevated IOP, normal-appearing optic discs, intact neuroretinal rims and peripapillary RNFLs, normal VF test results (defined as a pattern standard deviation within 95% confidence intervals), and glaucoma hemifield test results within normal limits^34^. Glaucoma was defined as the occurrence of glaucomatous optic nerve head changes (e.g. vertical cup-to-disc ratio>0.7, focal or diffuse neural rim loss, disc haemorrhage, or RNFL defects on red-free photography) and compatible glaucomatous VF defects^35^, regardless of the IOP level. Glaucomatous VF defects were defined as glaucoma hemifield test results outside the normal limits, pattern standard deviation with significance at P < 5%, and the presence of a cluster of three or more adjacent non-edge points in typical glaucomatous locations that did not cross the horizontal meridian, all of which were depressed on the pattern deviation plot at P < 5%, and one of which was depressed at P < 1%^13^. The subjects were divided into the following two groups: a highly myopic group (spherical equivalent ≤ −6.00 dioptres [D]) and a nonhighly myopic group (spherical equivalent > −6.00 D and ≤ − 0.5 D)^36^.

Exclusion criteria were known retinal or optic nerve disease; history of ocular trauma or ocular surgery, including cataract, refractive, and glaucoma surgery; and signal strength of ≤6 (of 10). Eyes that were diagnosed with pigment dispersion glaucoma, pseudoexfoliative glaucoma, or primary angle-closure glaucoma were also excluded. If both eyes of an enrolled patient met all inclusion and exclusion criteria, one eye was randomly chosen for the study.

### Standard automated perimetry

Standard automated perimetry VF tests were completed using the Swedish Interactive Threshold Algorithm standard 24–2 (Humphrey Field Analyzer; Carl Zeiss Meditec) strategies. Only reliable tests with fixation losses ≤20%, false negatives ≤33%, and false positives ≤15% were included. VFs with rim or eyelid artefacts, evidence of inattention, fatigue effects, or abnormal results caused by a disease other than glaucoma were excluded. Only mean deviations (MDs) measured by reliable tests were used in this study. Glaucomatous hemifield VF defects in the superior or inferior hemifields were defined as VF defects according to the Anderson-Patella criteria.

### Optical coherence tomography

**Figure 2:**
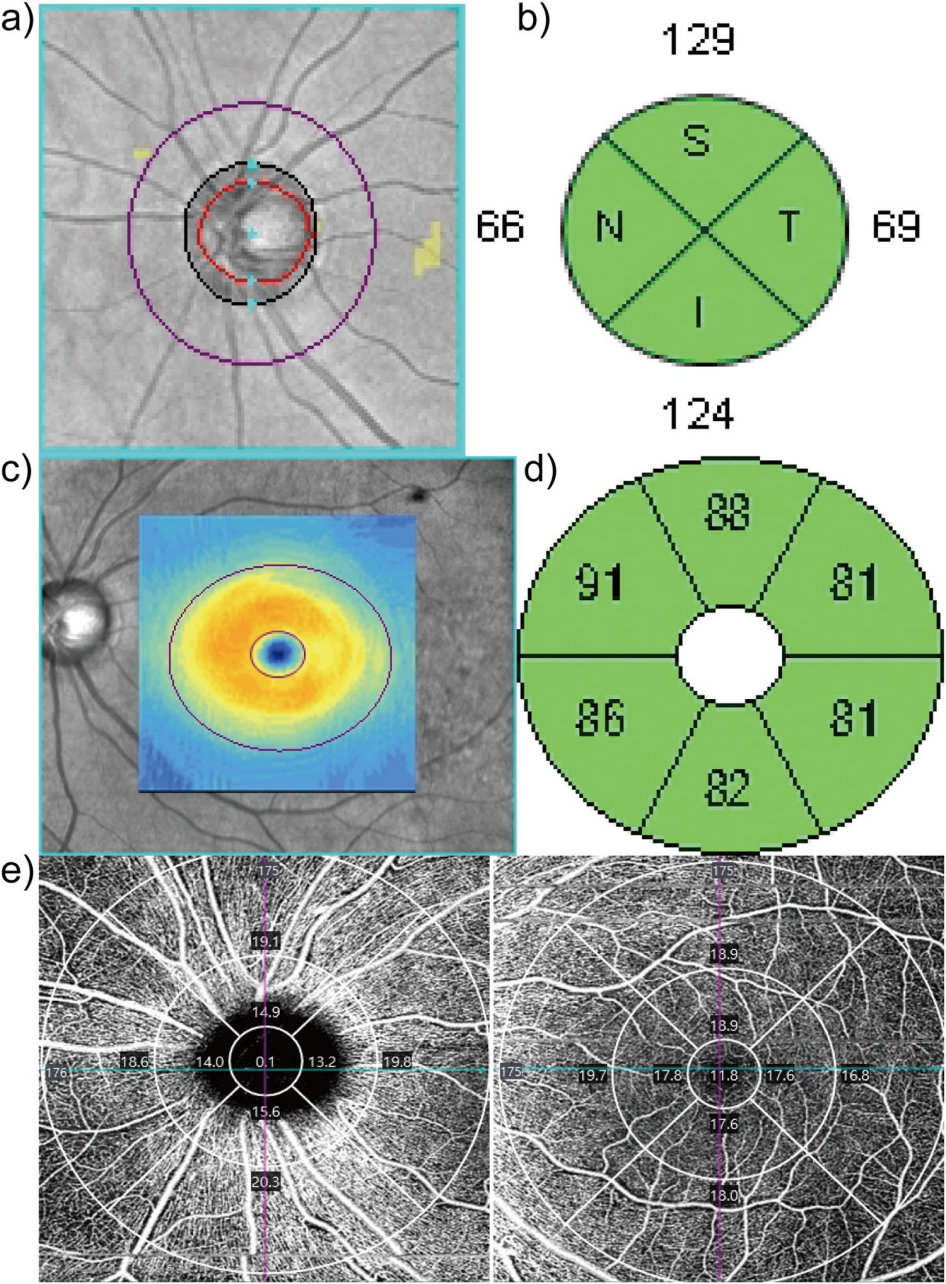
Comparison of the regions of interest for optical coherence tomography angiography (OCTA) and OCT measurements adopted in this study. (**a**) Optic disc scan with a 3.46  mm radius. (**b**) Retinal nerve fibre layer thickness of four sectors (superior, inferior, temporal, and nasal). (**c**) Macular scan with inner vertical and horizontal diameters of 1 and 1.2  mm, respectively, and outer vertical and horizontal diameters of 4 and 4.8  mm, respectively. (**d**) Ganglion cell-inner plexiform layer thickness of six sectors (superotemporal, superior, superonasal, inferotemporal, inferior, and inferonasal). (**e**) Peripapillary vessel density scan (left) and macular vessel density scan with Early Treatment Diabetic Retinopathy Study grid (right).

Macular GCIPL and peripapillary RNFL imaging with spectral-domain OCT were performed using the Cirrus HD-OCT macular and optic disc cube scans, respectively. The macular cube scan generated a GCIPL thickness map in a 6 × 6-mm^2^ area (512 × 128 pixels) centred at the fovea. Macular GCIPL thickness was measured within an annulus with inner vertical and horizontal diameters of 1 and 1.2  mm, respectively, and outer vertical and horizontal diameters of 4 and 4.8  mm, respectively. The optic disc cube scan generated an RNFL thickness map in a 6 × 6-mm^2^ area (200 × 200 pixels) centred at the optic disc. The circumpapillary RNFL thickness was measured in a circle of 3.46  mm in diameter (Fig. 2)^37^.

The following macular GCIPL thickness measurements were analysed: average, minimum, and sectoral (superonasal, superior, superotemporal, inferotemporal, inferior, and inferonasal). Regarding the peripapillary RNFL thickness, the average, superior, inferior, temporal, and nasal quadrant thicknesses were measured (Fig. 2). Among the OCT measurements, the results of average and inferior peripapillary RNFL thickness, and minimum and inferotemporal macular GCIPL thickness are included in the manuscript and other data are included in the supplemental table. The four variables were selected based on our preliminary data and other studies^36^. Images with a signal strength less than 6 on either the RNFL or macular scan, visible eye motion, blinking artefacts, or algorithm segmentation failure (i.e. on careful visual inspection of horizontal cross-sectional images on an output sheet, images with missing parts, misplacement of boundaries between retinal layers, or images showing seemingly distorted anatomy that resulted in readings of zero or otherwise abnormally low value), were excluded from the study^36^.

### Optical coherence tomography angiography

OCTA imaging was performed using the Cirrus HD-OCT 5000 and AngioPlex device with a wavelength of 840  nm and an A-scan rate of 68,000 scans per second. The volumetric scans were processed using optical microangiography (OMAG) algorithms to identify perfused vessels^38^. The OMAG algorithm analyses the changes in the phase and intensity information of the OCT scans to quantify motion contrast and then produces en face microvascular images of the superficial capillary plexus (SCP) [from the internal limiting membrane to the IPL] and the deep capillary plexus [from the inner nuclear layer to the outer plexiform layer (OPL)]^39^.

To investigate the peripapillary VD, OCTA was conducted using a 6 × 6-mm scan centred on the optic disc for data analysis. In the 6 × 6-mm scan pattern, there were 350 A-scans in each B-scan along the horizontal dimension, and 350 B-scans were repeated twice at each location. All scans were analysed using Cirrus OCTA software. VD (the total length of perfused vasculature per unit area in a region of measurement) of the SCP, according to the Early Treatment of Diabetic Retinopathy Study (ETDRS) subfields^40^, was automatically measured by the software. The diameters of the three concentric circles were 1, 3, and 6  mm, and each ring was divided into quadrants (Fig. 2). We analysed the peripapillary VD of the quadrants of the centre, inner ring, and outer ring, and the average VDs of the centre, inner ring, outer ring, and full area. Inner and outer ring measurements were analysed by sectoral locations (inferior, superior, nasal, and temporal). Regarding the macular VD, measurements were additionally analysed over the entire ETDRS 6-mm circle. In the manuscript, the results of the average value of each area and inferior inner/outer macular VD are demonstrated, and other data are added as supplemental data. Values with inaccurate boundaries identified on a manual review were excluded. Only scans of signal intensity ≥7 and without motion artefacts and segmentation errors were included in the analysis.

### Calculating new parameters for glaucoma detection

Preliminary data and other studies indicated that macular VD and disc VD of all areas are affected by signal strength^41^. Furthermore, according to our preliminary data, the outer macular VD was decreased in glaucomatous eyes, but the inner macular VD was not (Supplementary Fig. S1). Therefore, we calculated the average VD ratio (VDR), defined as average outer macular VD/average inner macular VD. Superior VDR was calculated as superior outer macular VD/average inner macular VD, and inferior VDR was calculated as inferior outer macular VD/average inner macular VD. These parameters were proposed to minimise the effect of variable parameters, such as signal length that could be associated with OCTA measurements.

### Statistics

Demographic variables between normal and glaucomatous eyes and between highly myopic eyes and nonhighly myopic eyes were analysed by the Student’s t-test and chi-square test. The average values of peripapillary RNFL thickness, macular GCIPL thickness, peripapillary VD, macular VD were compared using the Student’s t-test. We also used linear regression to adjust for demographic factors that were different between nonhighly and highly myopic eyes, when comparing OCT and OCTA measurements between nonhighly and highly myopic eyes. To compare the diagnostic ability of OCT measurements, including peripapillary RNFL thickness and macular GCIPL thickness, and OCTA measurements, including peripapillary VD and macular VD, in both the highly and nonhighly myopic groups, the AUROC was calculated. To obtain the confidence intervals for AUROC and sensitivities at 90% specificity, a bootstrap resampling procedure was used (n = 2000 resamples). For comparing the diagnostic abilities of the parameters, the AUROCs were compared by using DeLong *et al*.’s method^15^. All statistical analyses were performed using the R software, version 3.5.3 (R Foundation for Statistical Computing, Vienna, Austria). P values less than 0.05 were considered statistically significant.

## Supplementary information

Supplemental tables and figure legend.

## Data Availability

Data will be available only on request due to privacy/ethical restrictions. The data that support the findings of this study are available on request from the corresponding author, HWB.
