# Supplementary material for: Diagnostic ability of vessel density measured by spectral-domain optical coherence tomography angiography for glaucoma in patients with high myopia
Source: Sci Rep. 2020 Feb 20;10:3027. doi: 10.1038/s41598-020-60051-0 (PMC7033250; doi:10.1038/s41598-020-60051-0)

The Supplementary Tables and Figure for

Diagnostic ability of vessel density measured by spectral-domain optical coherence tomography angiography for glaucoma in patients with high myopia

Kwanghyun Lee, Kyung Joo Maeng, Joo Yeon Kim, Heon Yang, Wungrak Choi, Sang Yeop Lee, Gong Je Seong, Chan Yun Kim, and Hyoung Won Bae*

Department of Ophthalmology, Severance Hospital, Institute of Vision Research, Yonsei University College of Medicine, Seoul, Korea

2 tables and 1 figure

Supplemental table 1 peripapillary vessel density and macular vessel density obtained by OCT angiography and peripapillary retinal nerve fiber layer thickness and macular ganglion cell-inner plexiform layer thickness obtained by OCT

	Nonhighly Myopic Eyes (n=109)			Highly myopic eyes (n=60)			p†	P‡
	Normal (n=55)	Glaucoma (n=54)	P*	Normal (n=31)	Glaucoma (n=29)	P**		
Main RNFL thickness parameters (μm)								
Average	86.5 ± 10.4	69.9 ± 12.9	**<0.001**	85.5 ± 9.8	69.3 ± 8.9	**<0.001**	0.800	0.118
Superior	103.1 ± 18.5	86.6 ± 24.6	**<0.001**	97.7 ± 21.3	81.7 ± 17.5	**0.002**	0.163	**0.014**
Temporal	73.1 ± 13.9	58.4 ± 14.2	**<0.001**	79.6 ± 17.2	64.3 ± 14.1	**<0.001**	**0.016**	0.275
Inferior	106.2 ± 20.3	75.1 ± 19.0	**<0.001**	102.2 ±19.1	66.7 ± 13.4	**<0.001**	0.149	**0.023**
Nasal	63.1 ± 11.0	61.1 ± 15.9	0.439	61.8 ± 10.0	64.6 ± 10.6	0.302	0.556	0.503
Macular GCIPL parameters (μm)								
Average	79.0 ± 5.4	67.5 ± 8.0	**<0.001**	72.8 ± 5.7	65.1 ± 5.7	**<0.001**	**0.001**	**<0.001**
Minimum	74.5 ± 7.7	56.6 ± 9.4	**<0.001**	66.8 ± 10.8	54.8 ± 6.6	**<0.001**	**0.017**	**<0.001**
Superotemporal	77.4 ± 6.7	68.1 ± 10.9	**<0.001**	74.1 ± 7.7	67.8 ± 9.2	**0.005**	0.265	**0.027**
Superior	79.5 ± 6.6	70.8 ± 10.9	**<0.001**	73.4 ± 7.1	70.9 ± 8.1	0.196	**0.032**	**0.002**
Superonasal	82.9 ± 5.3	73.9 ± 11.1	**<0.001**	75.5 ± 8.2	71.6 ± 8.9	0.080	**0.002**	**<0.001**
Inferotemporal	77.5 ± 8.1	60.9 ± 10.6	**<0.001**	72.9 ± 6.7	57.0 ± 7.8	**<0.001**	**0.037**	**0.004**
Inferior	76.1 ± 7.2	61.9 ± 9.1	**<0.001**	68.0 ± 9.3	58.9 ± 6.3	**<0.001**	**0.001**	**<0.001**
Inferonasal	81.1 ± 8.3	68.4 ± 10.5	**<0.001**	72.5 ± 7.0	64.2 ± 8.0	**<0.001**	**<0.001**	**<0.001**
Vessel density – optic disc scan (mm^{-1})								
Average – Full	16.5 ± 2.2	15.2 ± 2.2	**0.003**	15.8 ± 3.7	13.6 ± 3.7	**0.025**	**0.046**	**0.001**
Average – Outer	17.4 ± 2.3	15.9 ± 2.4	**0.002**	16.3 ± 4.1	14.1 ± 4.0	**0.042**	**0.017**	**<0.001**
Superior Outer	17.9 ± 2.3	16.5 ± 2.7	**0.004**	16.8 ± 3.4	14.9 ± 4.3	0.070	**0.019**	**<0.001**
Temporal Outer	18.2 ± 3.2	16.7 ± 3.3	**0.015**	17.5 ± 4.6	14.6 ± 4.8	**0.020**	0.057	**0.016**
Inferior Outer	17.5 ± 2.5	15.0 ± 3.4	**<0.001**	16.2 ± 4.1	12.8 ± 4.6	**0.004**	**0.015**	**<0.001**
Nasal Outer	16.6 ± 2.9	16.1 ± 2.9	0.366	14.6 ± 4.6	15.0 ± 4.1	0.704	**0.019**	**0.002**
Average – Inner	15.5 ± 3.2	14.3 ± 2.8	**0.049**	15.7 ± 3.6	13.4 ± 4.1	**0.022**	0.615	0.108
Superior Inner	16.1 ± 3.9	15.2 ± 3.0	0.189	17.1 ± 3.9	14.8 ± 4.0	**0.026**	0.606	0.594
Temporal Inner	13.7 ± 4.8	12.9 ± 4.7	0.368	13.6 ± 5.4	11.6 ± 5.2	0.163	0.420	0.095
Inferior Inner	16.2 ± 3.3	12.3 ± 4.3	**<0.001**	16.1 ± 3.8	13.2 ± 5.3	**0.019**	0.571	0.555
Nasal Inner	16.8 ± 3.1	15.2 ± 3.9	**0.023**	16.1 ± 4.2	15.4 ± 4.4	0.532	0.772	0.204
Average - Centre	2.5 ± 2.8	2.1 ± 2.4	0.414	4.2 ± 4.2	2.5 ± 2.7	0.063	0.051	**0.023**
Vessel density – macular scan (mm^{-1})								
Average – Full	17.1 ± 1.8	15.5 ± 2.2	**<0.001**	17.3 ± 1.5	14.9 ± 2.6	**<0.001**	0.504	0.071
Average – Outer	17.4 ± 1.7	15.6 ± 2.4	**<0.001**	17.6 ± 1.6	14.8 ± 2.7	**<0.001**	0.494	0.054
Superior Outer	17.6 ± 2.0	16.2 ± 3.0	**0.004**	18.0 ± 1.8	16.2 ± 2.8	**0.004**	0.618	0.438
Temporal Outer	15.6 ± 2.8	14.3 ± 3.1	**0.018**	15.6 ± 2.3	13.3 ± 3.6	**0.006**	0.321	0.240
Inferior Outer	16.9 ± 2.2	13.7 ± 3.3	**<0.001**	17.3 ± 2.2	11.9 ± 3.8	**<0.001**	0.304	**0.031**

Nasal Outer	19.1 ± 1.6	18.0 ± 2.3	**0.003**	19.4 ± 1.7	17.5 ± 2.7	**0.003**	0.801	**0.030**
Average Inner	17.0 ± 2.2	16.9 ± 2.1	0.754	17.3 ± 1.6	16.3 ± 2.5	0.085	0.732	0.376
Superior Inner	17.4 ± 2.5	17.2 ± 2.6	0.691	17.6 ± 1.9	17.2 ± 2.6	0.451	0.677	0.760
Temporal Inner	16.7 ± 2.9	16.2 ± 2.7	0.324	16.6 ± 2.1	15.5 ± 3.7	0.152	0.418	0.321
Inferior Inner	16.8 ± 2.6	16.3 ± 2.5	0.382	17.5 ± 1.7	14.8 ± 3.5	**0.001**	0.420	0.153
Nasal Inner	17.2 ± 2.6	17.4 ± 2.5	0.697	17.4 ± 2.0	17.5 ± 2.8	0.912	0.737	0.601
Average Centre	8.2 ± 3.2	8.2 ± 3.1	0.939	8.9 ± 2.9	8.1 ± 2.9	0.329	0.508	0.920
Average VDR	1.0 ± 0.1	0.9 ± 0.1	**<0.001**	1.0 ± 0.1	0.9 ± 0.1	**<0.001**	0.305	**0.035**
Inferior VDR	1.0 ± 0.2	0.8 ± 0.2	**<0.001**	1.0 ± 0.1	0.7 ± 0.2	**<0.001**	0.133	**0.016**
Superior VDR	1.0 ± 0.1	1.0 ± 0.1	**<0.001**	1.0 ± 0.1	1.0 ± 0.1	0.059	0.347	0.875

RNFL: retinal nerve fiber layer, GCIPL: Ganglion cell-inner plexiform layer, Average VDR: average outer macular vessel density/average inner macular vessel density, Inferior VDR: inferior outer macular vessel density/ average inner macular vessel density

The data are given as mean (SD)

* Value for multiple comparison of normal eyes and glaucoma in highly myopic group.

** Value for multiple comparison of normal eyes and glaucoma in nonhighly myopic group.

† Value for multiple comparison of highly myopic and nonhighly myopic groups (t-test).

‡ Values for multiple comparison of highly myopic and nonhighly myopic groups after adjusting for age (linear regression)

Supplemental table 2 AUROC curve values in nonhighly myopic eyes and highly myopic eyes normal and glaucomatous eyes

	Nonhighly Myopic Eyes (n=109)		Highly myopic eyes (n=60)		P
	AUC (95% CI)	Sensitivity at 90% Specificity (%)	AUC (95% CI)	Sensitivity at 90% Specificity (%)	
Main RNFL thickness parameters (μm)					
Average	0.831 (0.754-0.907)	61.1 (44.4-75.9)	0.887 (0.805-0.969)	63.7 (43.3-87.3)	0.217
Superior	0.711 (0.610-0.812)	49.1 (31.8-64.4)	0.740 (0.614-0.867)	43.0 (13.8-70.9)	0.722
Temporal	0.768 (0.681-0.856)	44.0 (27.8-59.3)	0.763 (0.640-0.885)	44.8 (3.4-73.8)	0.941
Inferior	0.866 (0.796-0.936)	68.5 (46.9-86.1)	0.927 (0.866-0.988)	80.0 (46.7-96.7)	0.095
Nasal	0.587 (0.479-0.695)	22.2 (8.8-39.2)	0.577 (0.430-0.724)	17.2 (3.4-34.76)	0.082
Macular GCIPL parameters (μm)					
Average	0.890 (0.831-0.950)	68.8 (44.1-84.9)	0.819 (0.714-0.923)	58.6 (38.4-79.3)	0.245
Minimum	0.917 (0.863-0.971)	77.8 (56.1-93.8)	0.855 (0.755-0.956)	48.5 (10.0-87.3)	0.318
Superotemporal	0.754 (0.661-0.858)	49.1 (25.9-69.4)	0.697 (0.560-0.835)	42.1 (6.9-65.6)	0.505
Superior	0.747 (0.650-0.843)	50.0 (31.5-71.0)	0.582 (0.434-0.729)	20.9 (3.4-48.3)	0.069
Superonasal	0.752 (0.660-0.845)	53.7 (38.9-68.5)	0.631 (0.487-0.775)	27.6 (3.4-58.6)	0.167
Inferotemporal	0.881 (0.812-0.949)	77.8 (59.3-88.9)	0.925 (0.848-1.000)	90.0 (73.3-100.0)	0.490
Inferior	0.885 (0.816-0.951)	77.8 (51.4-90.3)	0.840 (0.733-0.948)	57.1 (5.3-93.1)	0.508
Inferonasal	0.842 (0.768-0.917)	63.9 (48.3-77.8)	0.774 (0.659-0.890)	46.2 (19.7-72.4)	0.333
Vessel density – optic disc scan (mm-1)					
Average Full	0.706 (0.606-0.806)	26.1 (5.6-55.6)	0.712 (0.576-0.848)	31.7 (0.0-70.0)	0.861
Average Outer	0.705 (0.604-0.806)	25.9 (6.6-54.1)	0.694 (0.556-0.833)	16.7 (0.0-66.7)	0.998
Superior Outer	0.664 (0.560-0.768)	28.7 (5.6-61.1)	0.645 (0.502-0.788)	34.5 (3.4-62.1)	0.840
Temporal Outer	0.696 (0.596-0.796)	25.9 (3.7-48.2)	0.723 (0.587-0.858)	21.6 (0.0-72.4)	0.758
Inferior Outer	0.746 (0.652-0.841)	37.0 (9.3-66.7)	0.760 (0.634-0.887)	41.4 (0.1-73.5)	0.862
Nasal Outer	0.555 (0.446-0.664)	20.4 (0.0-40.7)	0.517 (0.367-0.667)	5.7 (0.0-31.0)	0.450
Average Inner	0.631 (0.523-0.738)	16.9 (0.0-40.7)	0.669 (0.531-0.808)	33.9 (3.3-60.0)	0.626
Superior Inner	0.621 (0.513-0.729)	9.3 (0.0-22.2)	0.712 (0.580-0.844)	31.0 (0.0-62.1)	0.297
Temporal Inner	0.554 (0.445-0.663)	13.0 (4.7-24.1)	0.609 (0.465-0.753)	20.7 (0.0-48.3)	0.550
Inferior Inner	0.762 (0.671-0.853)	35.2 (14.8-59.3)	0.671 (0.532-0.811)	34.5 (10.3-55.3)	0.289
Nasal Inner	0.649 (0.546-0.753)	20.4 (3.7-40.7)	0.546 (0.397-0.605)	17.2 (0.0-41.4)	0.267
Average Centre	0.559 (0.449-0.670)	20.5 (9.4-33.8)	0.624 (0.481-0.768)	16.7 (5.0-40.0)	0.402
Vessel density – macular scan (mm-1)					
Average Full	0.702 (0.603-0.800)	33.3 (18.5-48.2)	0.763 (0.643-0.884)	46.7 (20.0-70.0)	0.323
Average Outer	0.731 (0.636-0.826)	37.0 (22.2-52.8)	0.809 (0.700-0.917)	50.0 (26.7-80.0)	0.206
Superior Outer	0.635 (0.531-0.740)	30.5 (16.7-44.4)	0.723 (0.590-0.857)	41.4 (10.3-69.0)	0.313
Temporal Outer	0.649 (0.545-0.753)	18.5 (1.9-38.9)	0.699 (0.565-0.833)	31.0 (13.8-55.2)	0.563
Inferior Outer	0.790 (0.704-0.877)	51.9 (35.2-69.0)	0.890 (0.809-0.972)	73.3 (48.7-93.3)	0.070

Nasal Outer	0.661 (0.557-0.764)	29.6 (14.8-48.2)	0.740 (0.611-0.870)	27.6 (10.3-52.8)	0.349
Average Inner	0.523 (0.414-0.633)	10.7 (0.0-27.8)	0.596 (0.450-0.743)	26.7 (6.7-50.0)	0.330
Superior Inner	0.514 (0.404-0.625)	20.4 (3.7-33.3)	0.497 (0.345-0.650)	20.8 (0.0-41.6)	0.905
Temporal Inner	0.576 (0.468-0.684)	13.0 (0.0-33.3)	0.560 (0.410-0.709)	20.7 (6.9-38.3)	0.863
Inferior Inner	0.566 (0.457-0.675)	13.0 (1.9-32.4)	0.757 (0.627-0.886)	60.0 (30.0-76.8)	**0.021**
Nasal Inner	0.539 (0.430-0.649)	11.1 (0.0-25.0)	0.567 (0.417-0.717)	14.5 (3.4-34.5)	0.768
Average Centre	0.505 (0.396-0.615)	9.3 (0.0-25.9)	0.558 (0.411-0.705)	20.0 (3.3-46.7)	0.458
Average VDR	0.852 (0.781-0.923)	65.5 (52.7-76.4)	0.909 (0.837-0.981)	73.3 (53.3-93.3)	0.272
Inferior VDR	0.820 (0.739-0.902)	59.3 (40.7-77.8)	0.941 (0.886-0.996)	80.0 (60.0-96.7)	**0.017**
Superior VDR	0.695 (0.596-0.794)	29.6 (16.7-44.4)	0.664 (0.518-0.811)	44.8 (17.3-69.0)	0.733

AUROC: Area Under the Receiver Operating Characteristics, RNFL: retinal nerve fiber layer, GCIPL: Ganglion cell-inner plexiform layer, Average VDR: average outer macular vessel density/average inner macular vessel density, Inferior VDR: inferior outer macular vessel density/ average inner macular vessel density

* calculated by delong's method.

Supplemental Figure Legend

Supplemental Figure 1. Boxplots illustrating the relationships between macular vessel density and signal strength. a) Outer macular vessel density and signal strength in nonhighly myopic eyes. b) Inner macular vessel density and signal strength in nonhighly myopic eyes. c) Outer macular vessel density and signal strength in highly myopic eyes. d) Inner macular vessel density and signal strength in highly myopic eyes

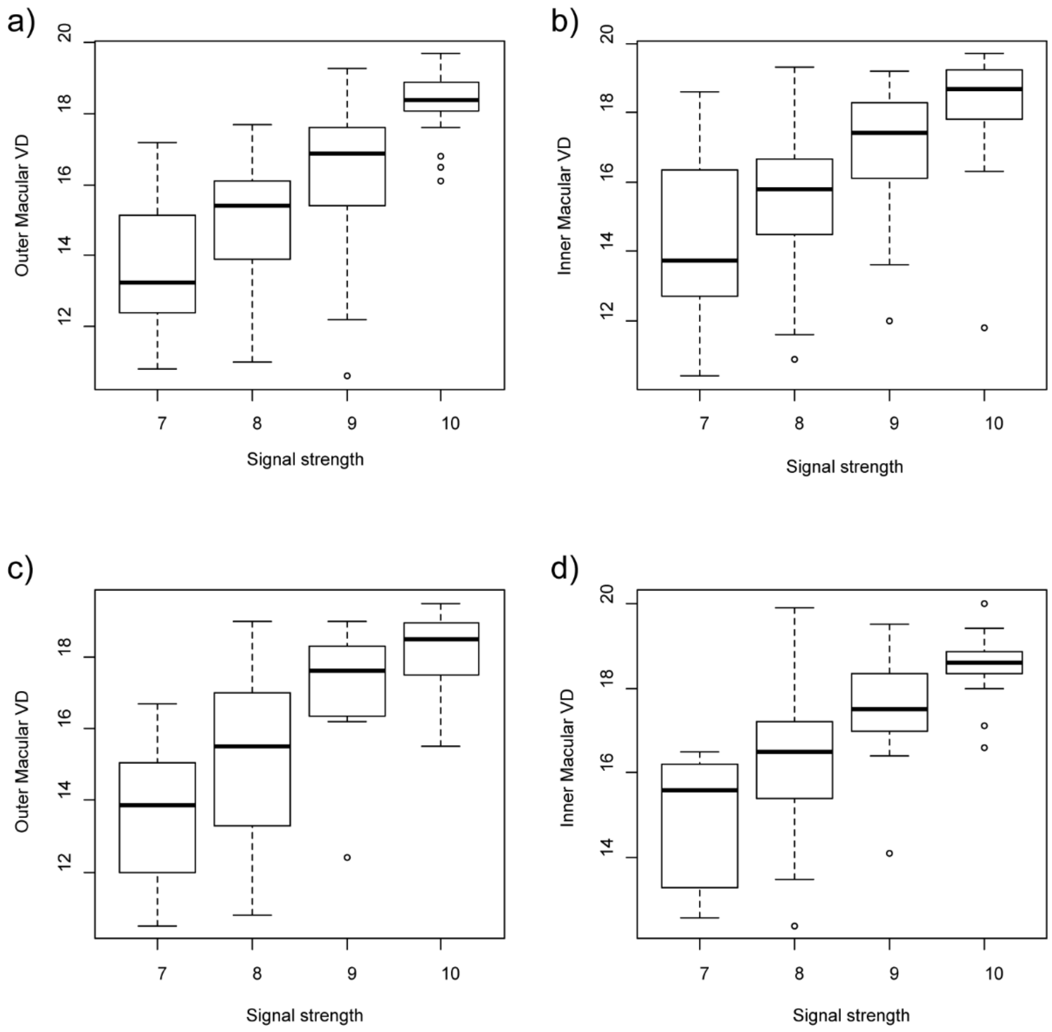

Supplement: Supplementary file 1 — Supplemental tables and figure legend. [file 41598_2020_60051_MOESM1_ESM.pdf]
